# Supplementary material for: Acylated Flavone O-Glucuronides from the Aerial Parts of Nepeta curviflora
Source: Molecules. 2020 Aug 20;25(17):3782. doi: 10.3390/molecules25173782 (PMC7504054; doi:10.3390/molecules25173782)

Acylated flavone *O*-glucuronides from the aerial parts of *Nepeta curviflora*

Maysaa Rabee, Øyvind M. Andersen, Torgils Fossen, Kjersti Hasle Enerstvedt, Hijazi Abu Ali and Saleh Rayyan

Supplementary data

NMR spectra

Table of content

- **Page 1:** Title page
- **Page 2:** Table of content
- **Page 3-10:** NMR and Mass spectra of compound **1**
- **Page 11:** High resolution mass spectrum of compound **1**.
- **Page 12:** Mass spectra of the non-methylated version of compound **1**.
- **Page 13-18:** NMR spectra of compound **2.**
- **Page 19:** High resolution mass spectrum of compound **2**.
- **Page 20:** Mass spectra of the non-methylated version of compound **2**.

Figure S1. 1D ^{1}H NMR spectrum of apigenin 7-*O*-(2''-*O*-(2'''-(*E*-caffeoyl)-β-glucuronopyranosyl)-β-glucuronopyranoside) (**1**)

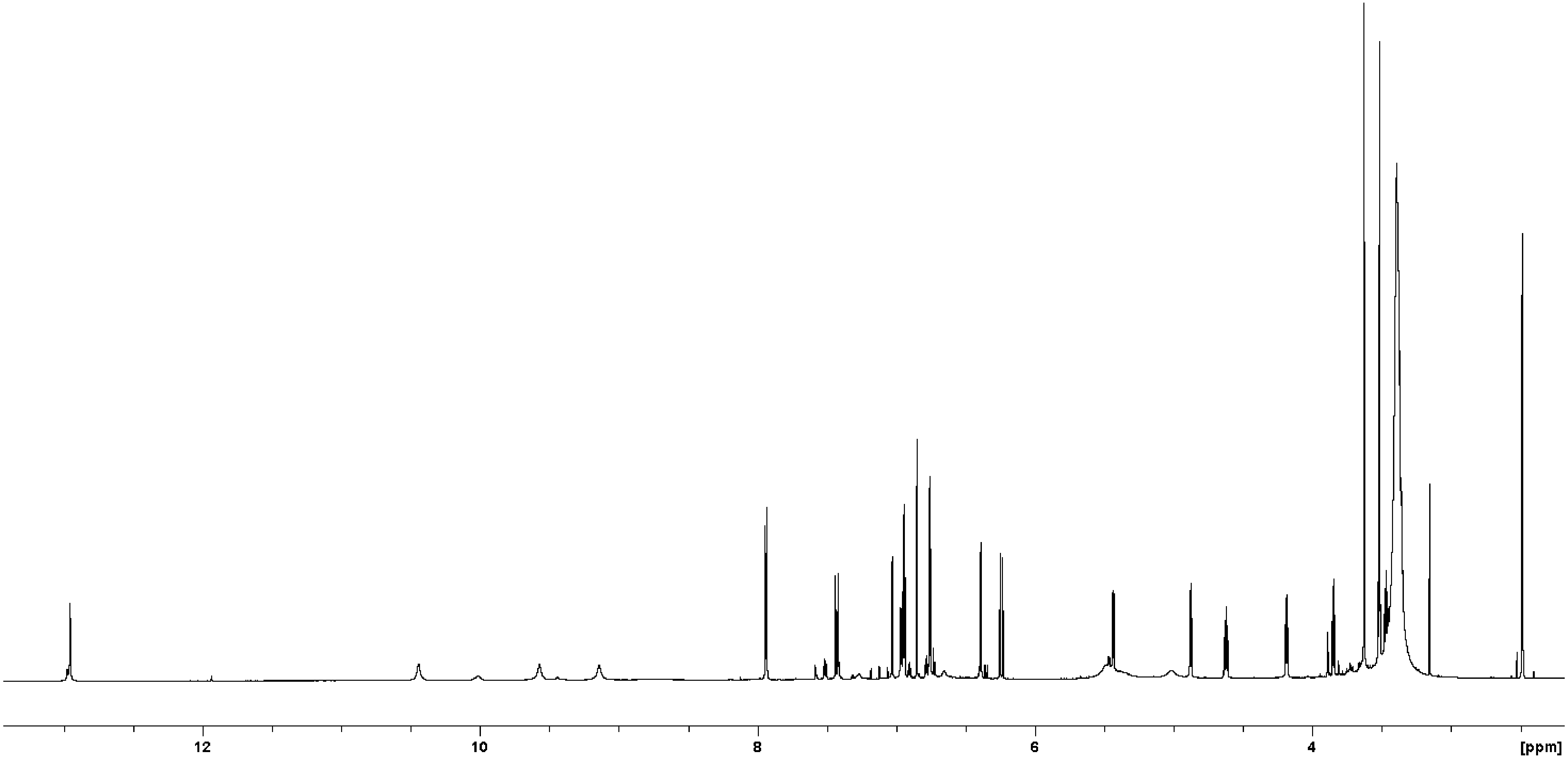

Figure S2. 1D ^{13}C CAPT NMR spectrum of apigenin 7-*O*-(2''-*O*-(2'''-(*E*-caffeoyl)-β-glucuronopyranosyl)-β-glucuronopyranoside) (**1**)

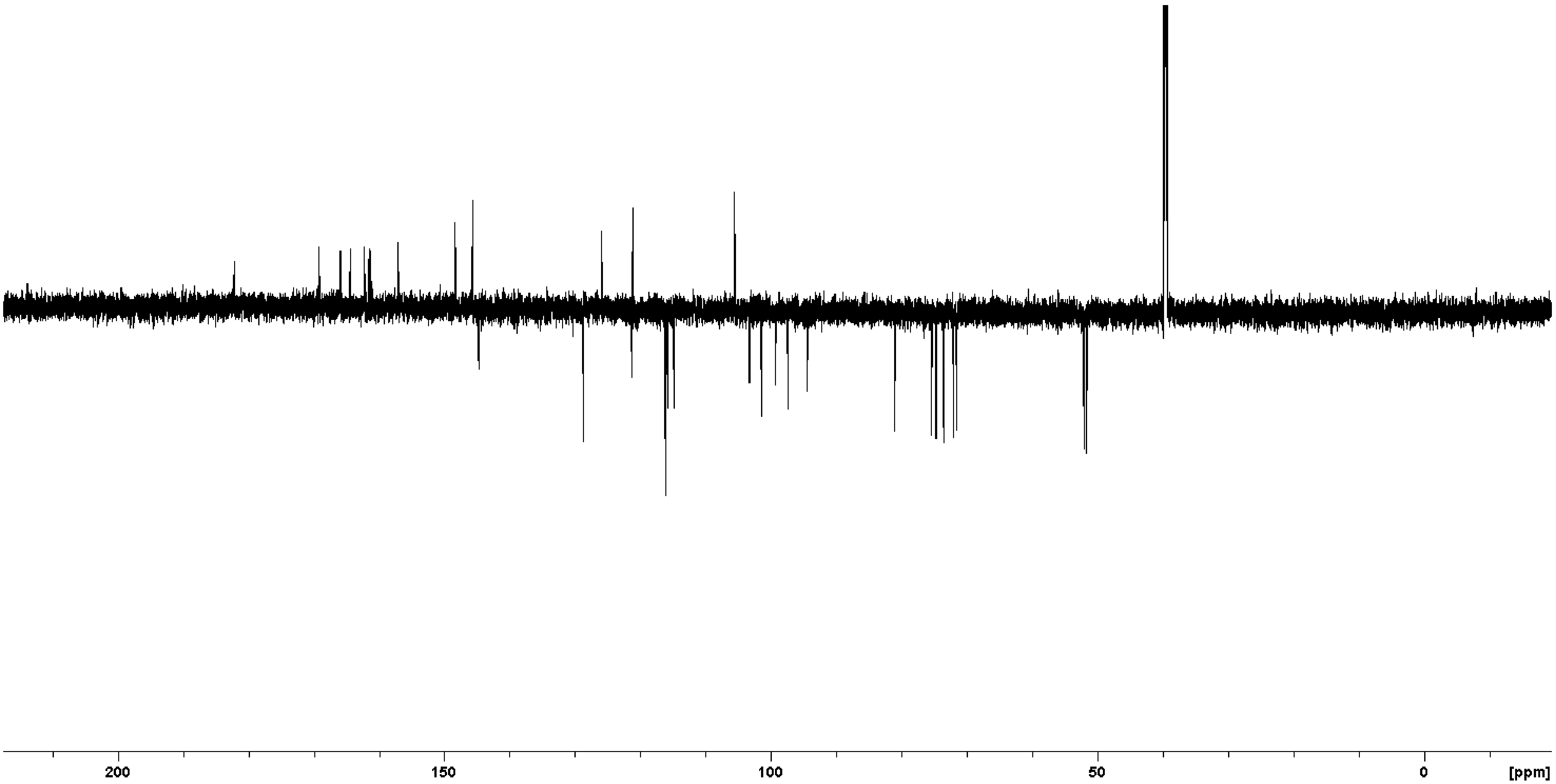

Figure S3. 2D ^{1}H-^{13}C edited HSQC NMR spectrum of apigenin 7-*O*-(2''-*O*-(2'''-(*E*-caffeoyl)-β-glucuronopyranosyl)-β-glucuronopyranoside) (**1**)

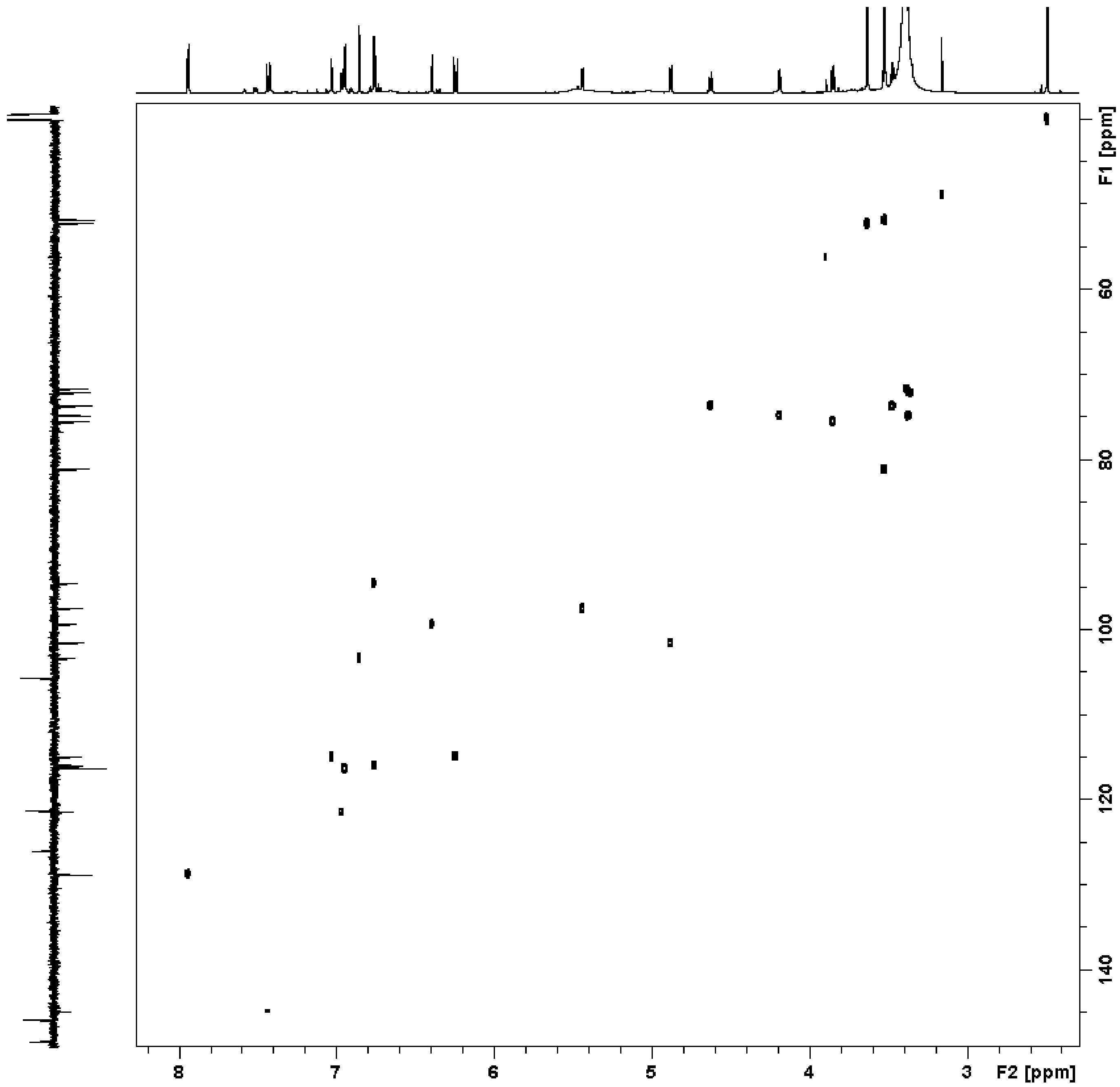

Figure S4. 2D ^{1}H-^{13}C HMBC NMR spectrum of apigenin 7-*O*-(2''-*O*-(2'''-(*E*-caffeoyl)-β-glucuronopyranosyl)-β-glucuronopyranoside) (**1**)

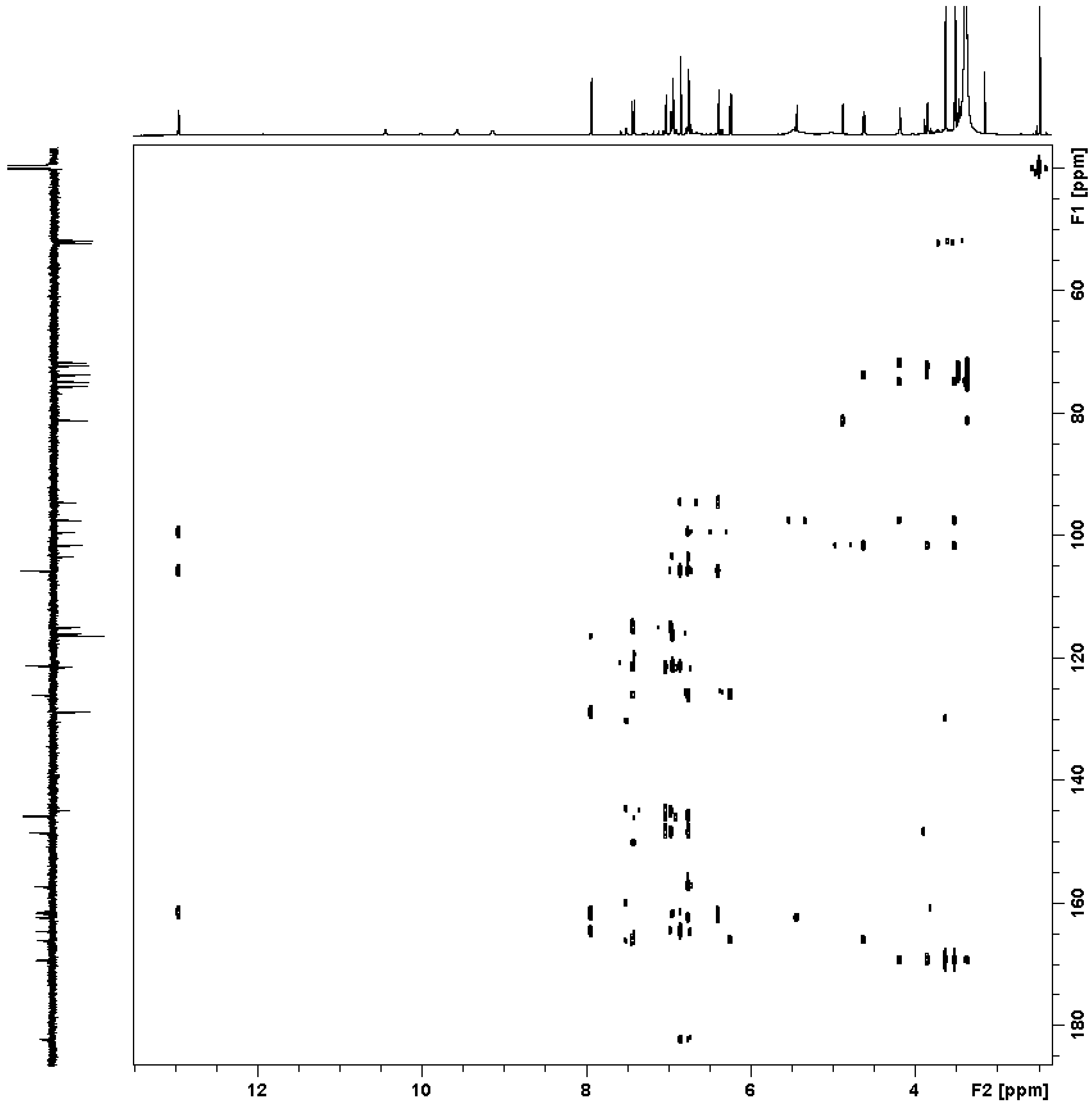

Figure S5. 2D ^{1}H-^{13}C HSQC-TOCSY NMR spectrum of apigenin 7-*O*-(2''-*O*-(2'''-(*E*-caffeoyl)-β-glucuronopyranosyl)-β-glucuronopyranoside) (**1**)

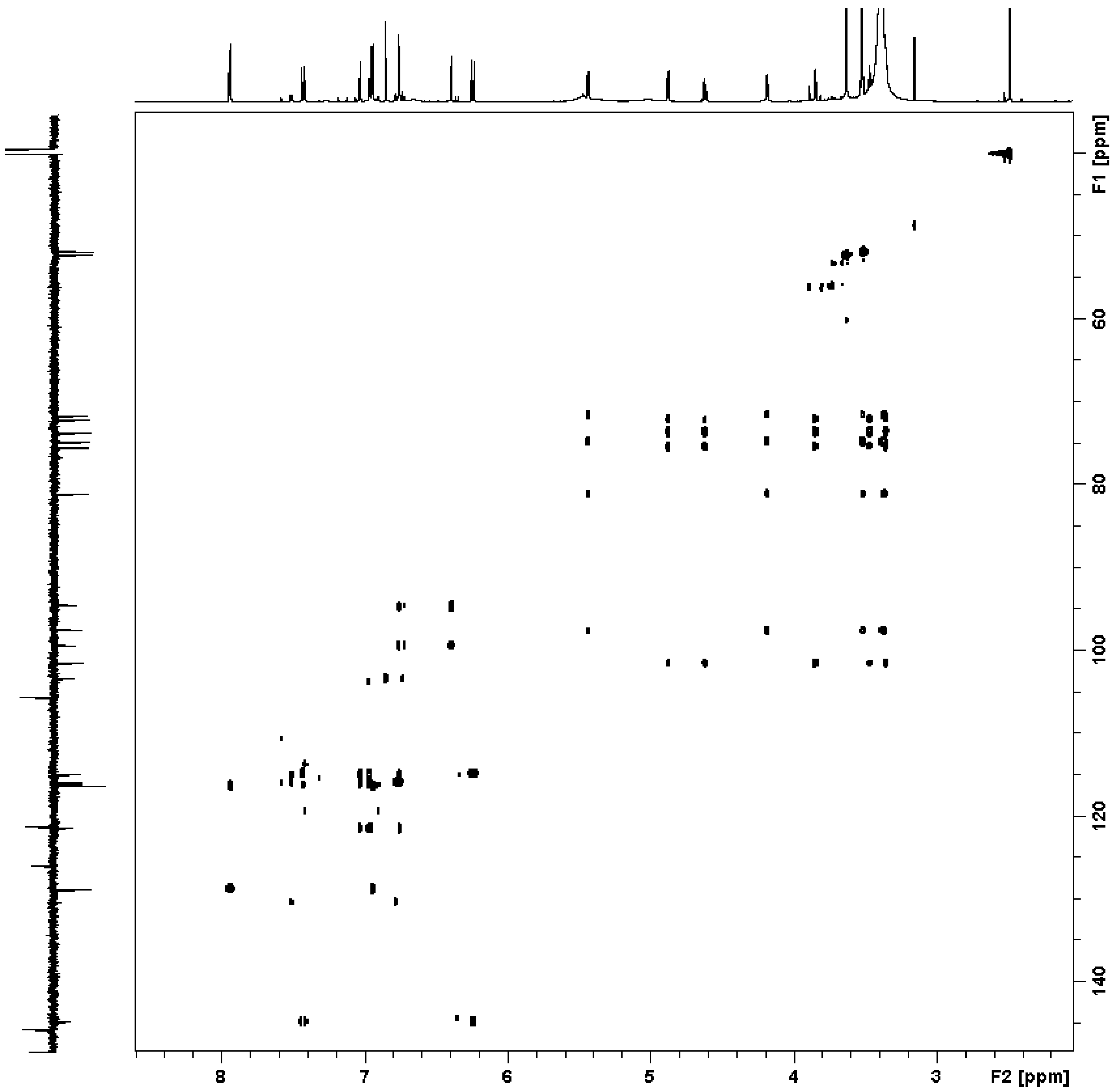

Figure S6. 2D ^{1}H-^{13}C H2BC NMR spectrum of apigenin 7-*O*-(2''-*O*-(2'''-(*E*-caffeoyl)-β-glucuronopyranosyl)-β-glucuronopyranoside) (**1**)

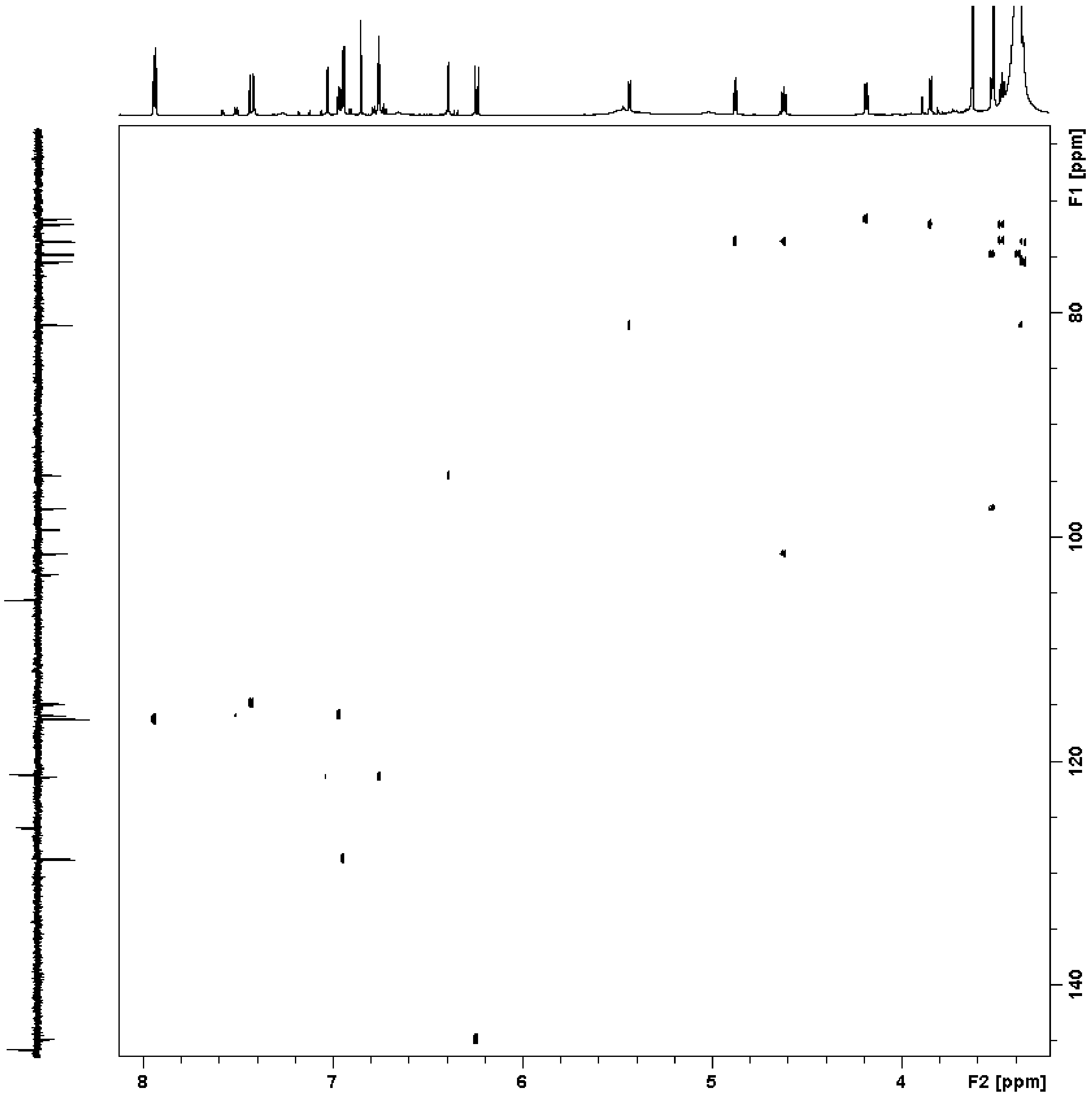

Figure S7. 2D ^1H-^1H COSY NMR spectrum of apigenin 7-*O*-(2''-*O*-(2'''-(*E*-caffeoyl)-β-glucuronopyranosyl)-β-glucuronopyranoside) (**1**)

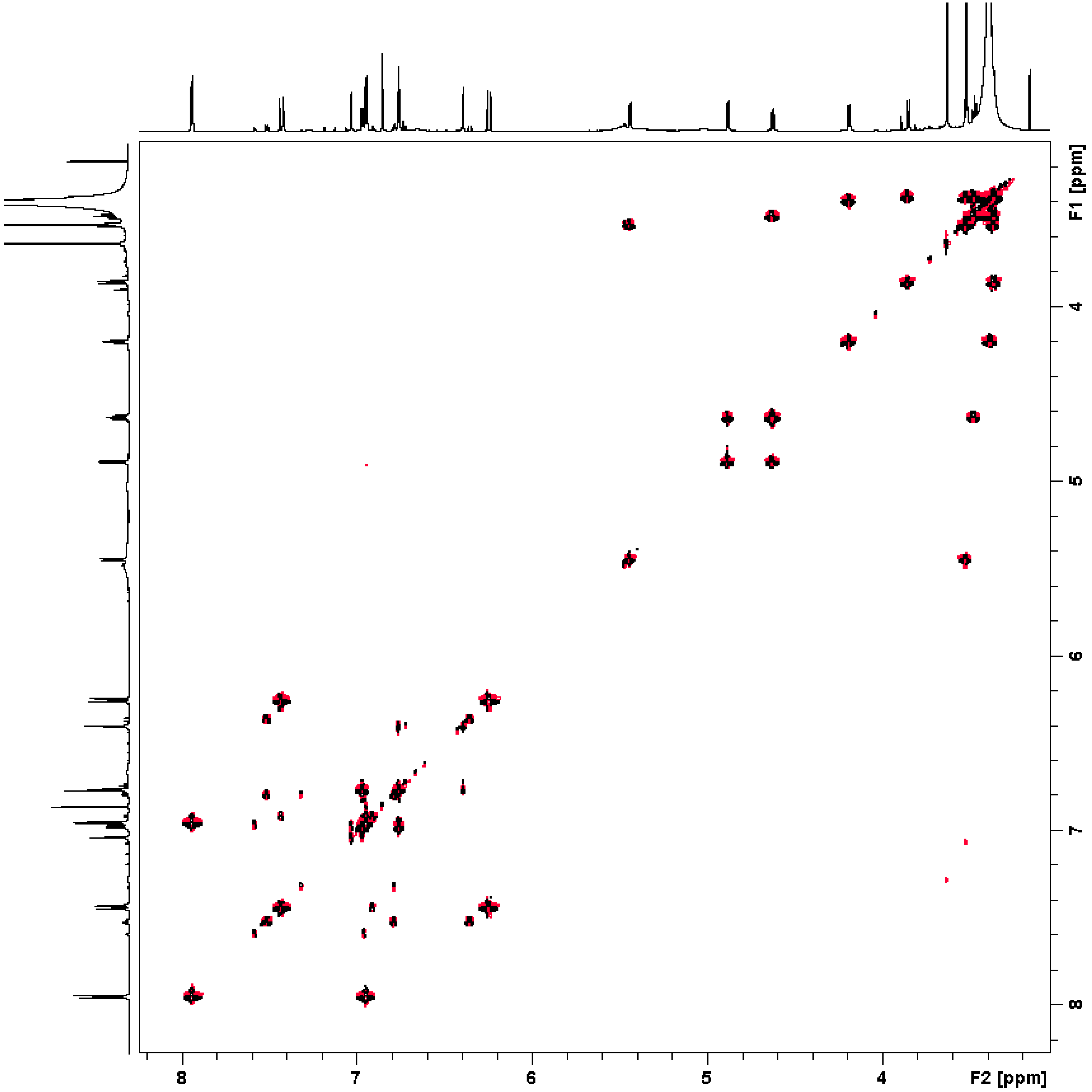

Figure S8. 2D ^{1}H-^{1}H ROESY NMR spectrum of apigenin 7-*O*-(2''-*O*-(2'''-(*E*-caffeoyl)-β-glucuronopyranosyl)-β-glucuronopyranoside) (**1**)

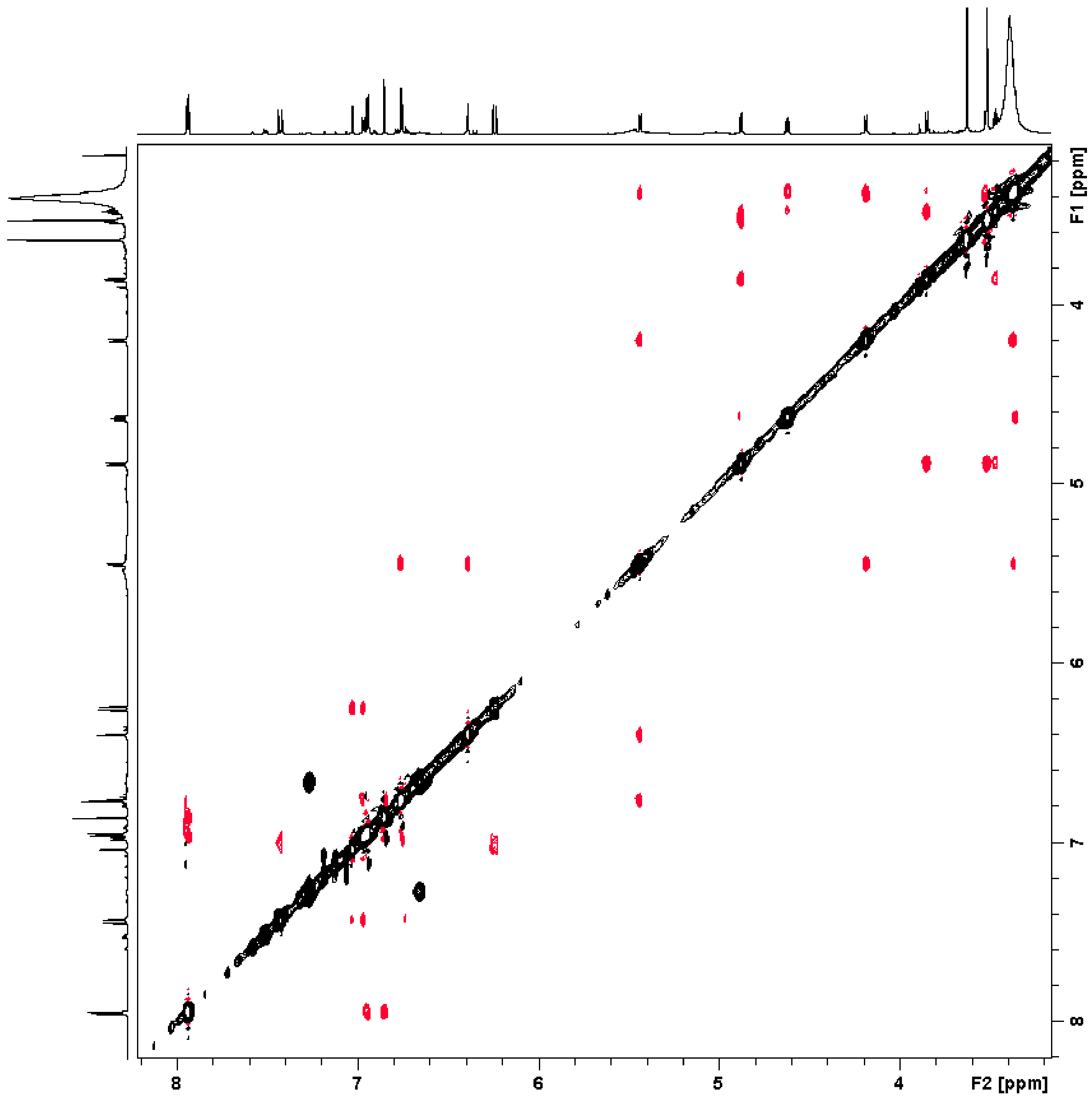

Figure S9. HR Mass spectrum of the dimethylester form of apigenin 7-*O*-(2''-*O*-(2'''-(*E*-caffeoyl)-β-glucuronopyranosyl)-β-glucuronopyranoside) (**1**)

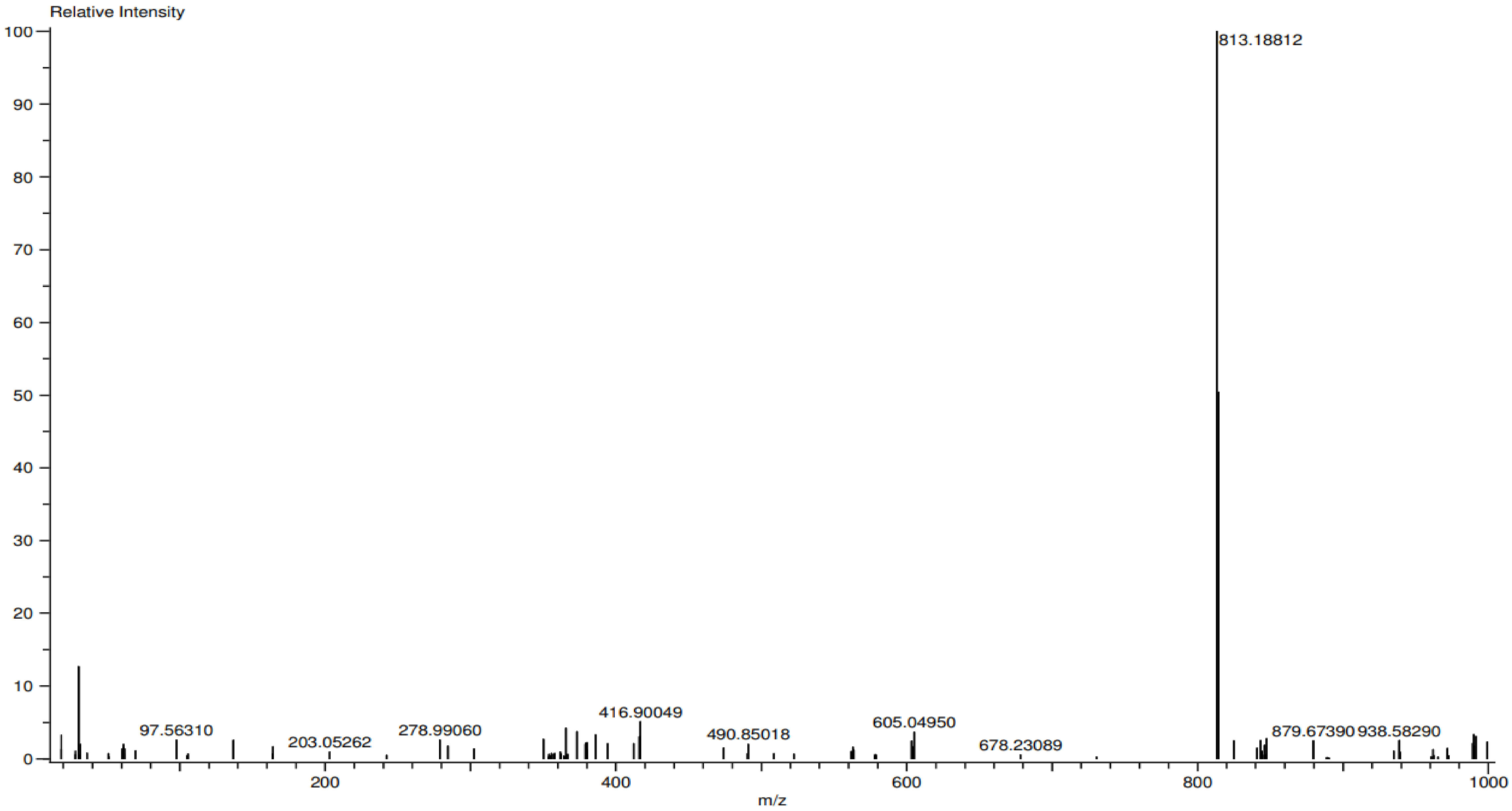

Figure S10. Mass spectrum of apigenin 7-*O*-(2''-*O*-(2'''-(*E*-caffeoyl)-β-glucuronopyranosyl)-β-glucuronopyranoside) (**1**)

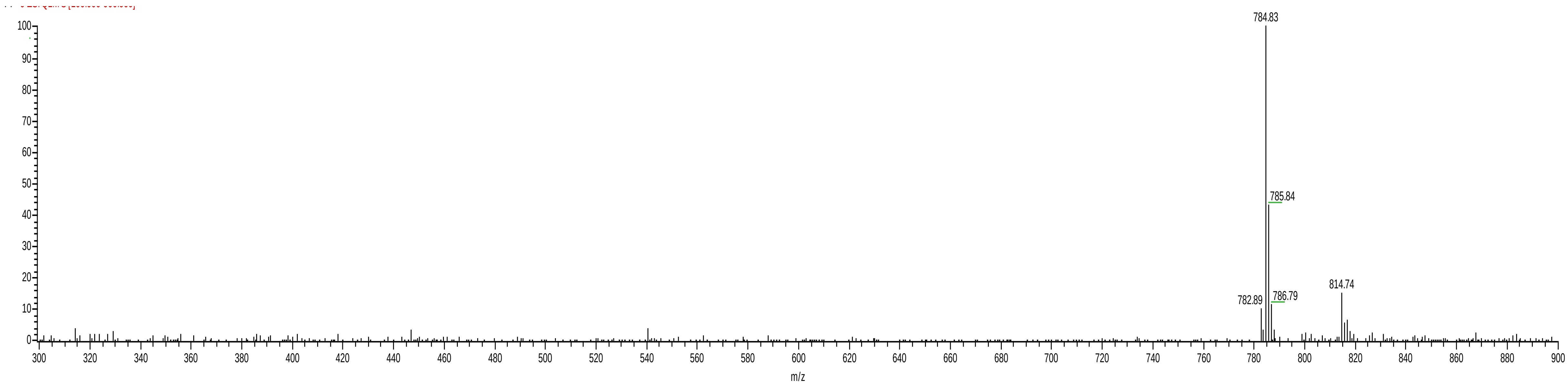

Figure S11. 1D ^{1}H NMR spectrum of luteolin 7-*O*-(2''-*O*-(2'''-(*E*-caffeoyl)-β-glucuronopyranosyl)-β-glucuronopyranoside) (**2**)

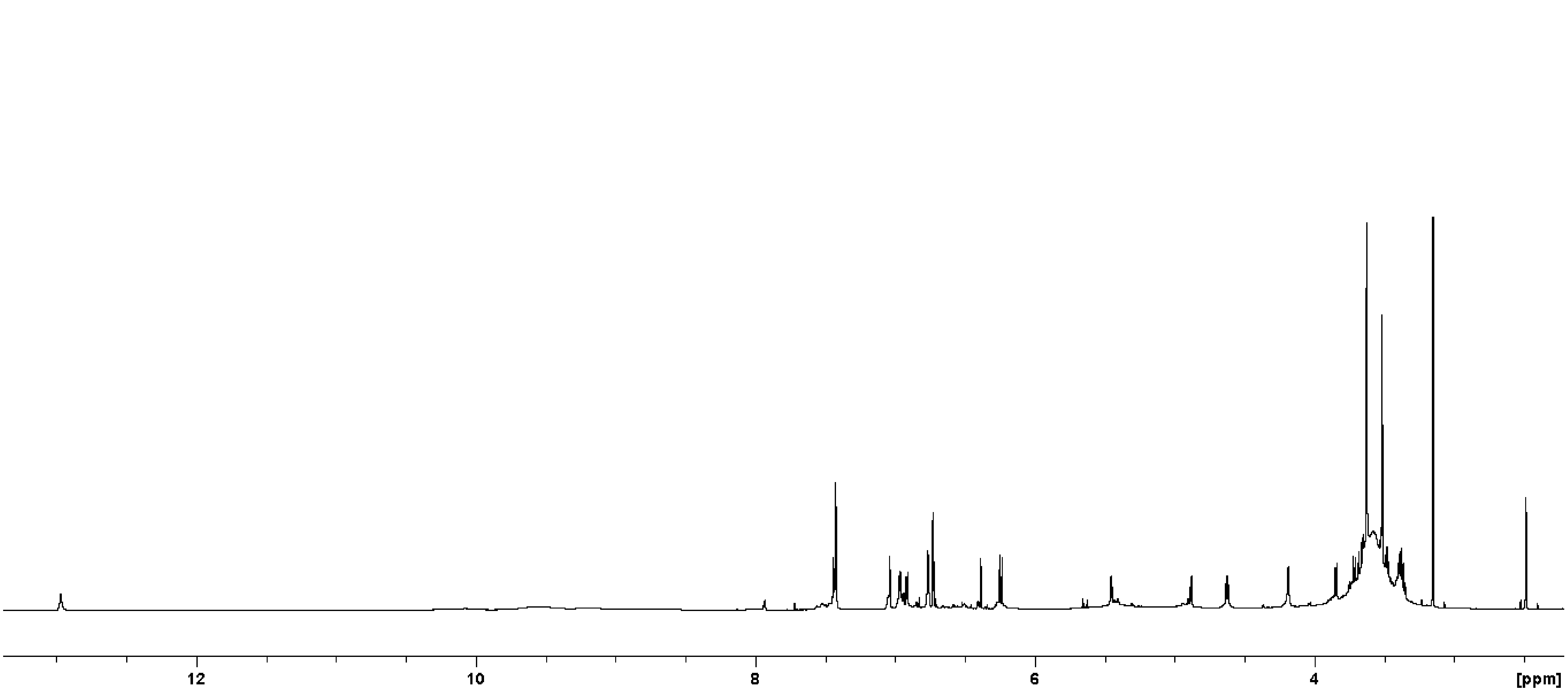

Figure S12. 1D ^{13}C CAPT NMR spectrum of luteolin 7-*O*-(2''-*O*-(2'''-(*E*-caffeoyl)-β-glucuronopyranosyl)-β-glucuronopyranoside) (**2**)

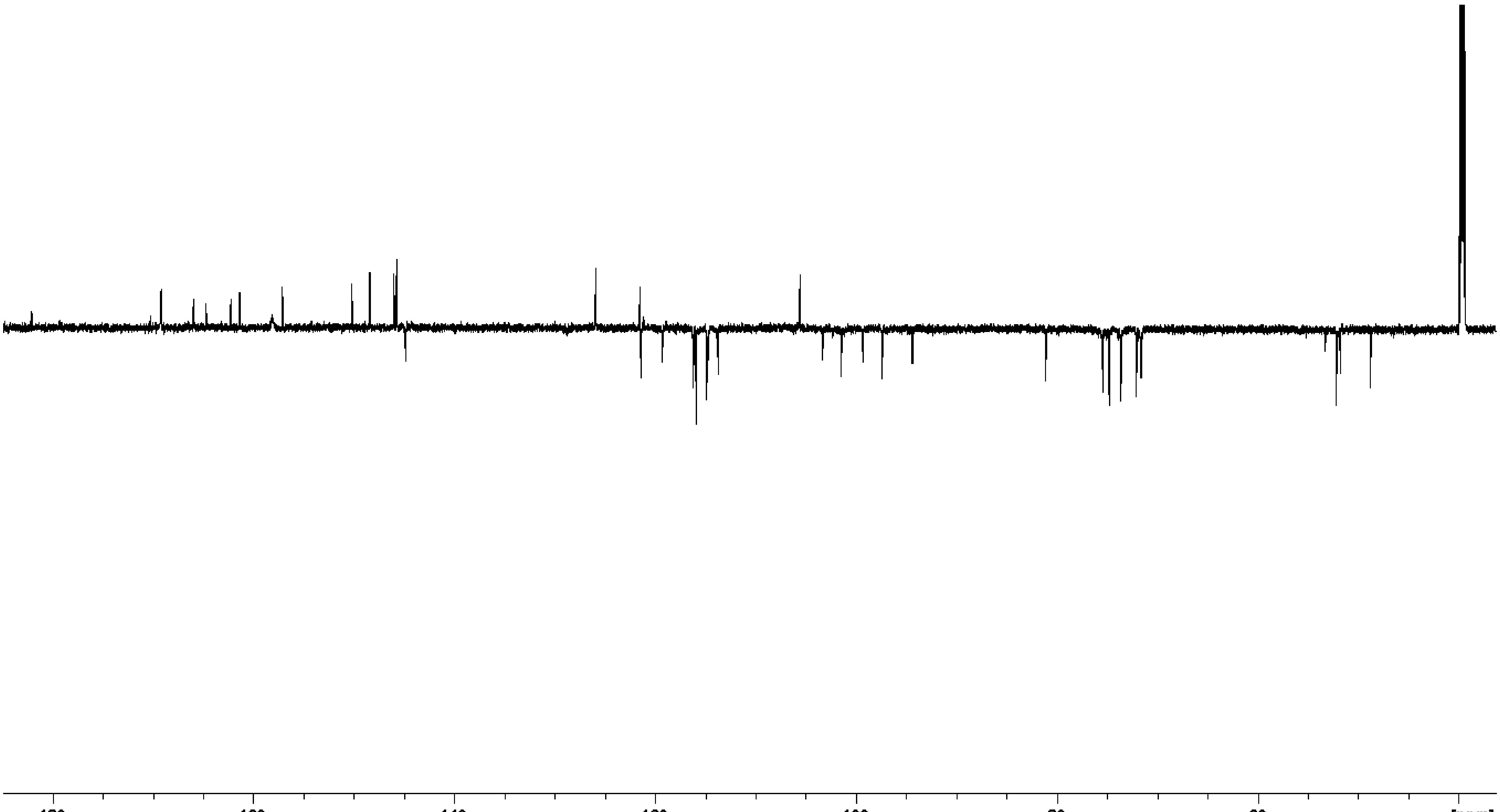

Figure S13. 2D ^{1}H-^{13}C HMBC NMR spectrum of luteolin 7-*O*-(2''-*O*-(2'''-(*E*-caffeoyl)-β-glucuronopyranosyl)-β-glucuronopyranoside) (**2**)

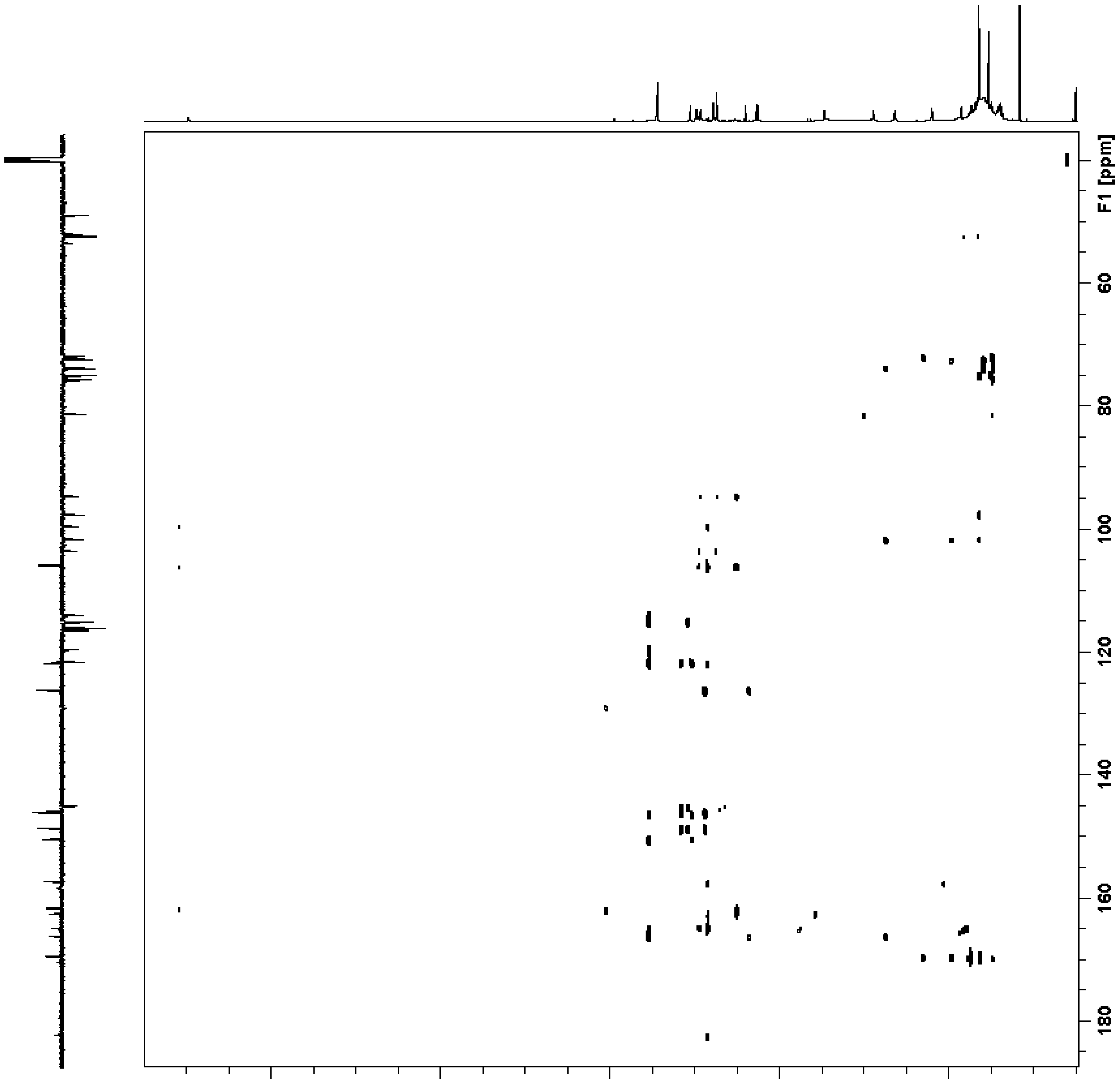

Figure S14. 2D ^{1}H-^{13}C edited HSQC NMR spectrum of luteolin 7-*O*-(2''-*O*-(2'''-(*E*-caffeoyl)-β-glucuronopyranosyl)-β-glucuronopyranoside) (**2**)

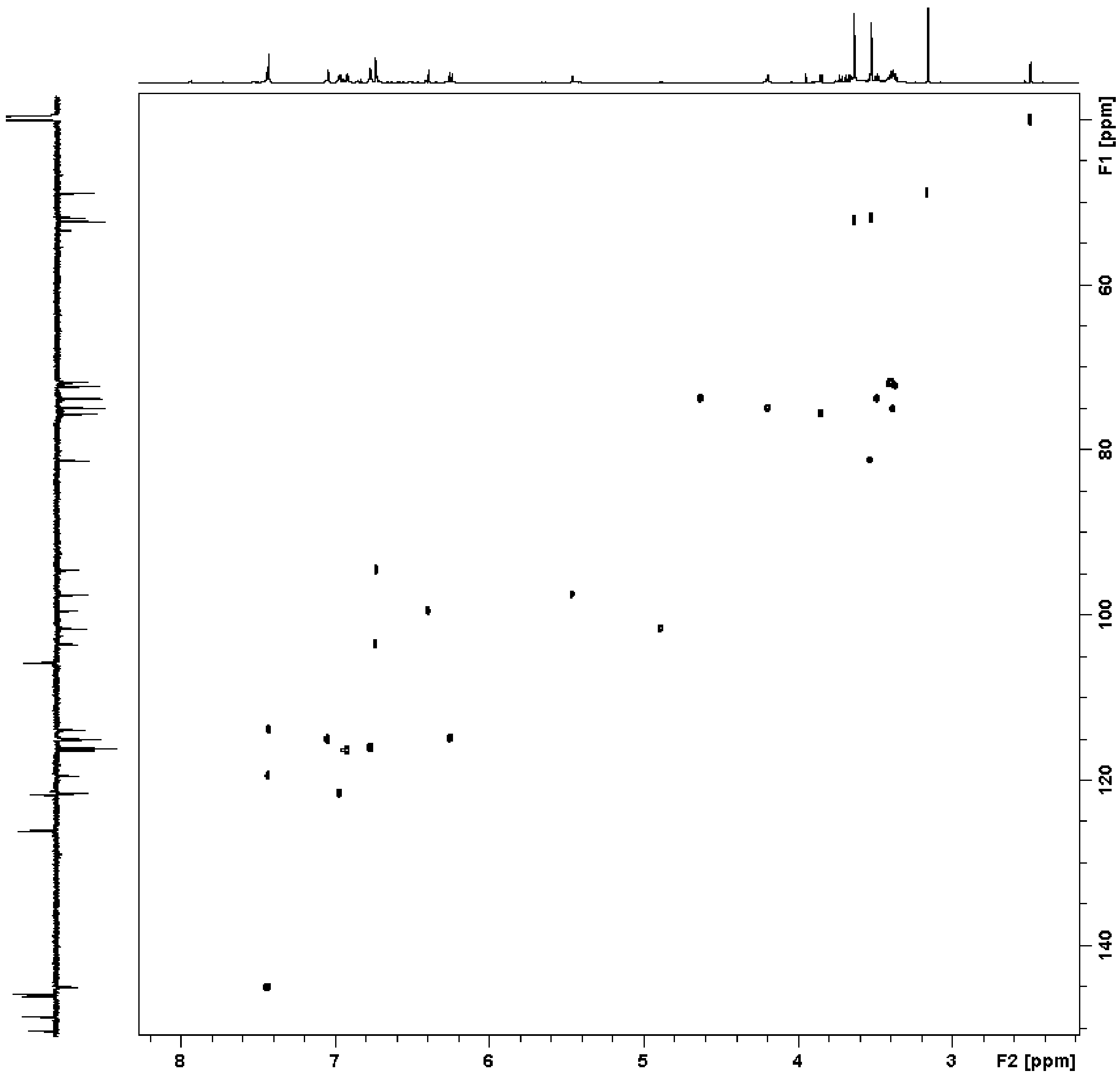

Figure S15. 2D ^{1}H-^{13}C H2BC NMR spectrum of luteolin 7-*O*-(2''-*O*-(2'''-(*E*-caffeoyl)-β-glucuronopyranosyl)-β-glucuronopyranoside) (**2**)

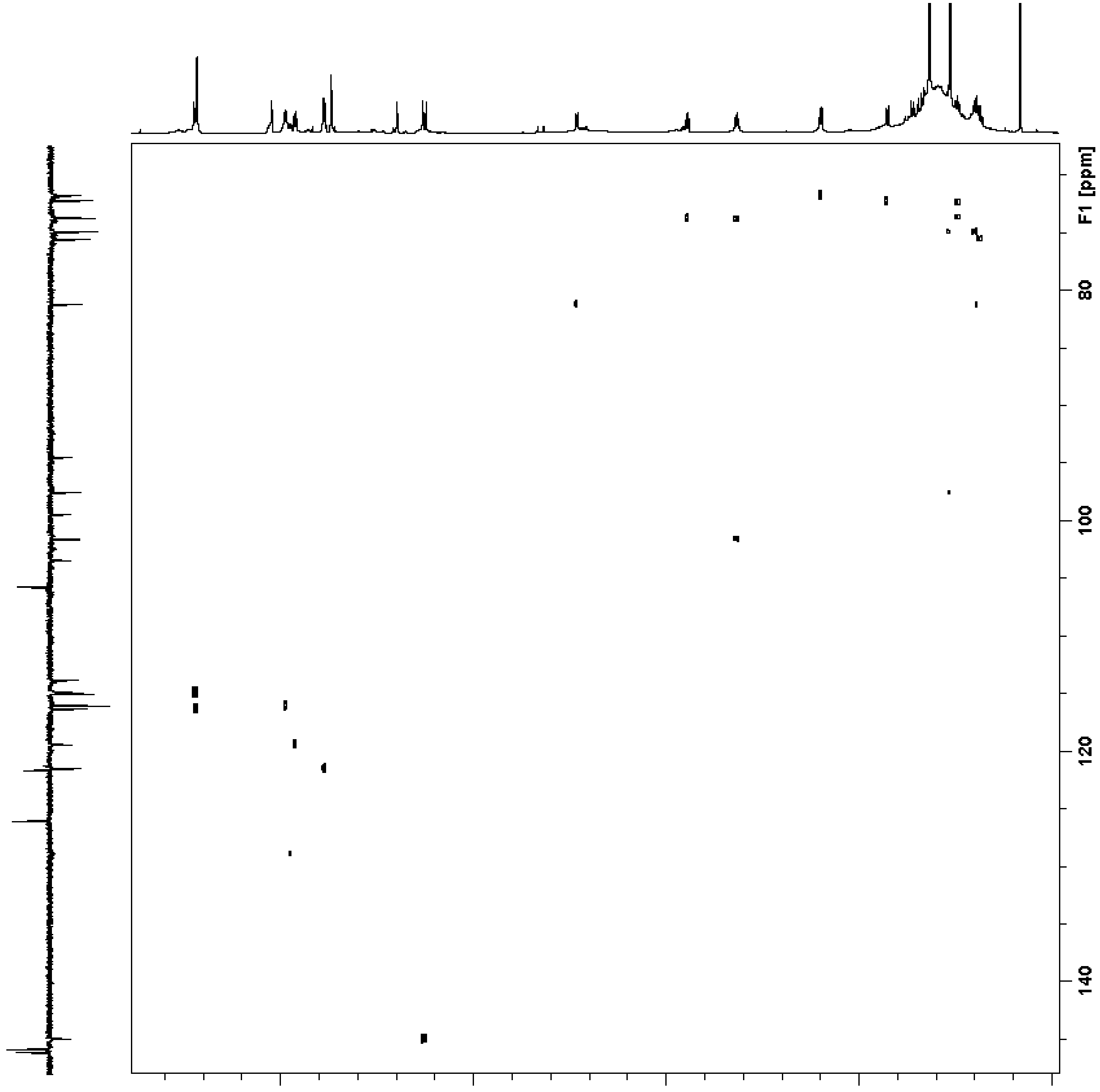

Figure S16. 2D ^1H-^1H COSY NMR spectrum of luteolin 7-*O*-(2''-*O*-(2'''-(*E*-caffeoyl)-β-glucuronopyranosyl)-β-glucuronopyranoside) (**2**)

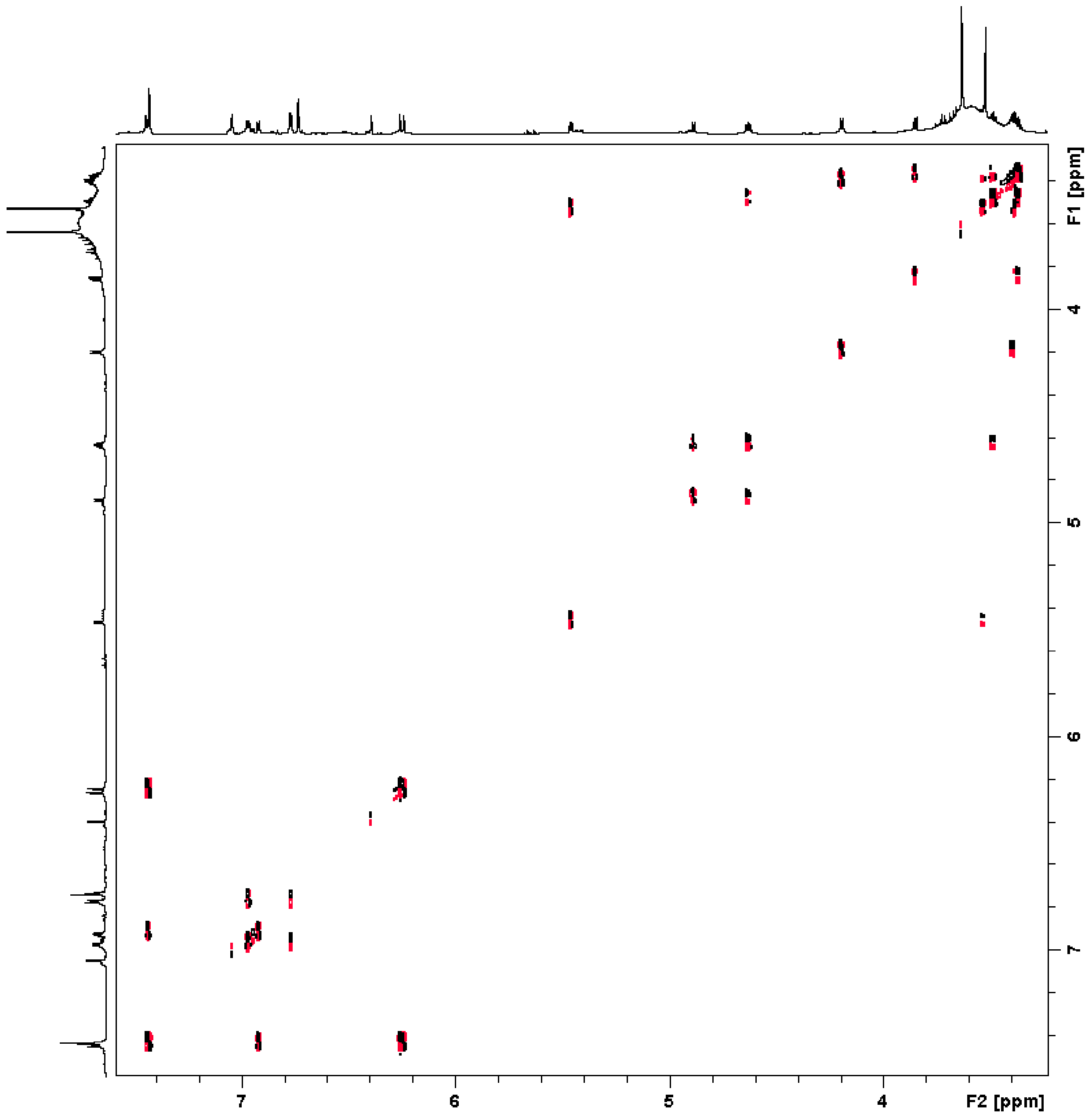

Figure S17. HR mass spectrum of the methyl ester form of luteolin 7-*O*-(2''-*O*-(2'''-(*E*-caffeoyl)-β-glucuronopyranosyl)-β-glucuronopyranoside) (**2**)

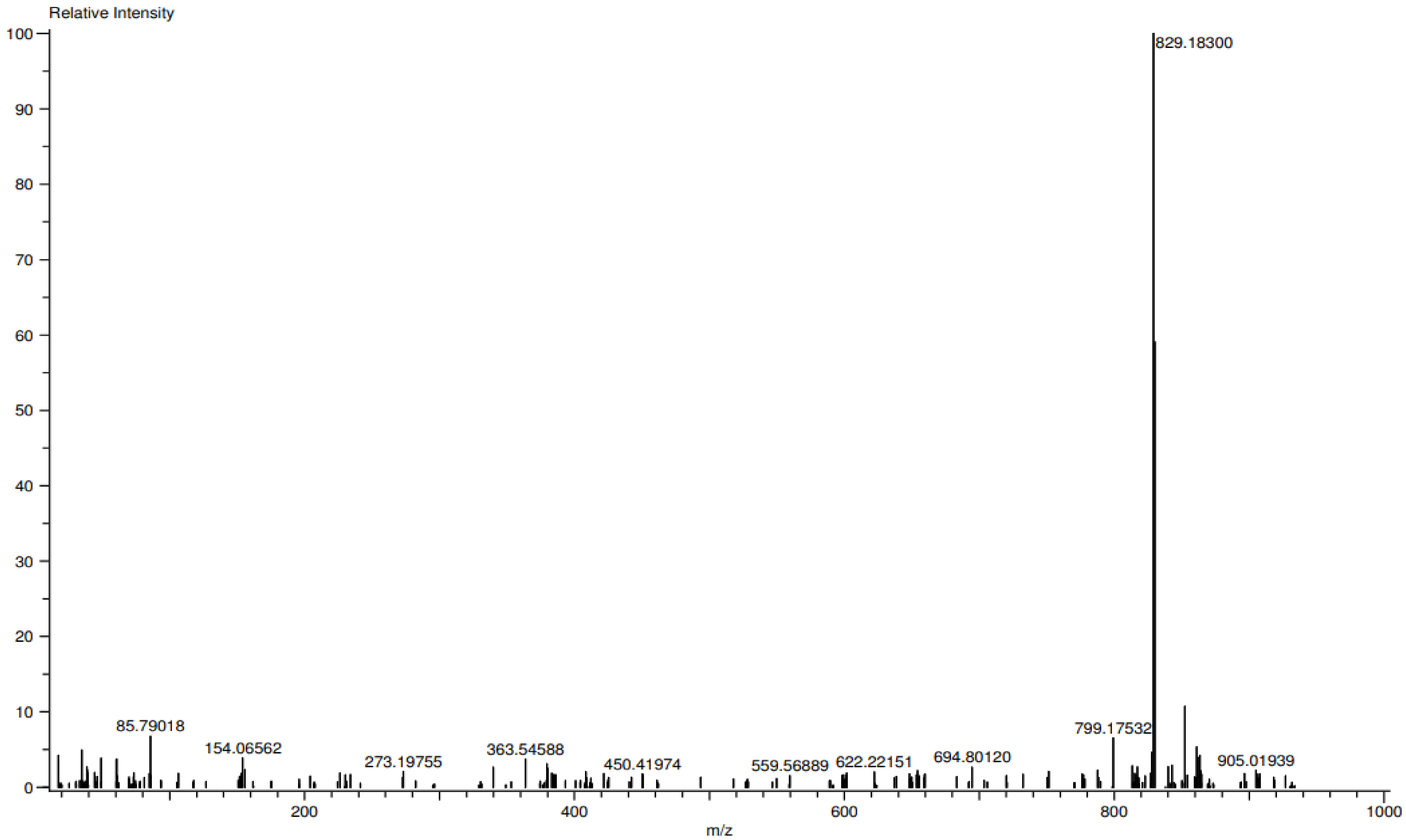

Figure S18. Mass spectrum of luteolin 7-*O*-(2''-*O*-(2'''-(*E*-caffeoyl)-β-glucuronopyranosyl)-β-glucuronopyranoside) (**2**)

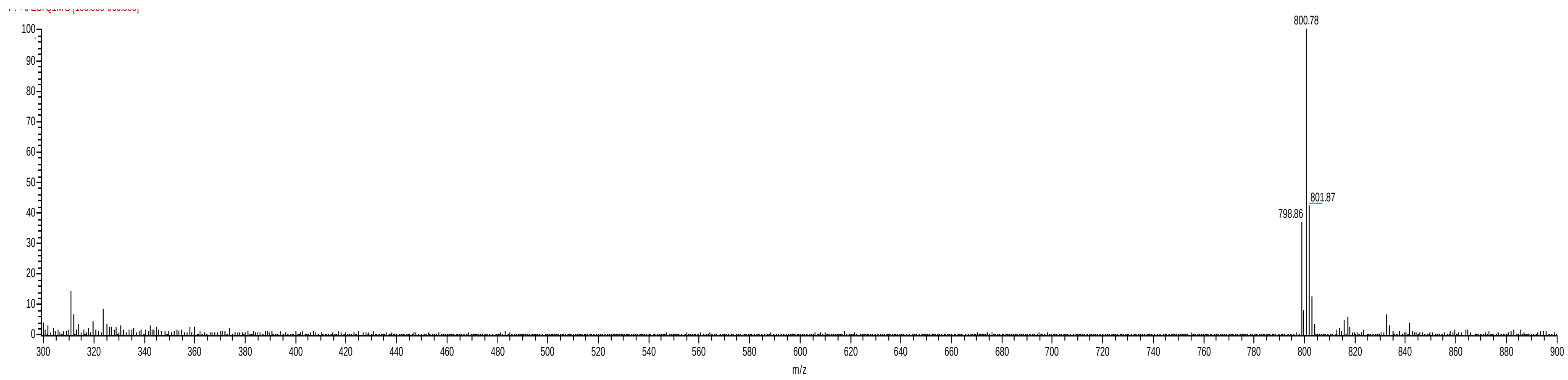

Supplement: Supplementary file 1 [file molecules-25-03782-s001.zip › Supplementary data MS and NMR.pdf]
