# Supplementary material for: Acylated Flavone O-Glucuronides from the Aerial Parts of Nepeta curviflora
Source: Molecules. 2020 Aug 20;25(17):3782. doi: 10.3390/molecules25173782 (PMC7504054; doi:10.3390/molecules25173782)
Supplement: Supplementary file 1 [file molecules-25-03782-s001.zip › Supplementary data_tables.pdf]

Table S1: Chromatographic (HPLC) and spectral (UV and MS) data recorded for **1–6** from *Nepeta curviflora***.**

Compound	t_R (min)	UV (λ_{max}) (nm)	Molecular mass $[MH]^+$	
			Observed	Calculated
**1**	31.5	332, 267, 220	813.1881	813.1878
**2**	29.6	344, 250, 209	829.1830	829.1827
**3**	31.5	330, 230		
**4**	14.0	330, 225		
**5**	34.2	337, 268		
**6**	25.7	338, 267		

Table S2: ^{1}H and ^{13}C spectral data (ppm) and coupling constants (Hz) for compounds **3** and **4** dissolved in DMSO-D6 at 25°C.

	**3 (^{1}H)**	**3 (^{13}C)**	**4 (^{1}H)**	**4 (^{13}C)**
C=O		166.07		166.07
α	6.26 *d* 15.9	113.03	6.26 *d* 15.9	113.03
β	7.48 *d* 15.9	146.53	7.48 *d* 15.9	146.53
1		125.46		125.46
2	7.06 *d* 2.2	115.10	7.10 *d* 2.2	115.10
3		144.30		144.30
4		148.91		148.91
5	6.77 *d* 8.2	115.94	6.77 *d* 8.2	115.94
6	7.01 *dd* 2.2, 8.2	121.91	7.07 *dd* 2.2, 8.2	121.91
C=O		170.11		
α	5.12 *dd* 2.2, 7.7	72.93		
β_A	2.95 *m*	36.35		
β_B	2.95 *m*			
1’		126.82		
2’	6.65 *d* 2.1	116.82		
3’		145.78		
4’		144.16		
5’	6.64 *d* 8.1	115.61		
6’	6.49 *dd* 2.1, 8.1	120.25		
OCH_3	3.63 *s*	52.15		

Table S3. ^{1}H and ^{13}C NMR chemical shift values of apigenin (**5**) and apigenin 7-*O*-β-glucuronopyranoside (**6**) in DMSO-D_6 at 298 K.

	**5** δ ^{1}**H**	**5** δ ^{13}**C**	**6** δ ^{1}**H**	**6** δ ^{13}**C**
2		163.6		164.38
3	6.77 *s*	102.9	6.85 *s*	103.22
4		181.9		182.11
5		161.6		161.23
6	6.18 *d* 2.1	98.9	6.44 *d* 2.2	99.64
7		164.2		163.07
8	6.48 *d* 2.1	94.0	6.82 *d* 2.2	94.96
9		157.3		157.06
10		103.8		105.46
1’		121.3		121.15
2’/6’	7.92 ‘*d*’ 8.9	128.6	7.94 ‘*d*’ 8.9	128.72
3’/5’	6.92 ‘*d*’ 8.9	116.1	6.93 ‘*d*’ 8.9	116.12
4’		161.3		161.48
5-OH	12.95 *s*		12.95 *s*	
7-OH	10.88 *s*			
4’-OH	10.38 *s*		10.40 *s*	
7-*O*-β-glucuronopyranoside				
1’’			5.06 *d* 7.7	100.03
2’’			3.26 *dd* 7.7, 9.0	73.22
3’’			3.30 *t* 9.0	76.56
4’’			3.18 *dd* 9.6, 9.0	69.68
5’’			3.44 *ddd* 2.1, 5.8, 9.6	77.29
6A’’			3.71 *dd* 2.1, 11.9	60.73
6B’’			3.48 *dd* 5.9, 11.9	

Table S4: Solvent composition and elution volumes used for separation of XAD-7 purified extract of aerial parts of *Nepeta curviflora* using a 100×5 cm Sephadex LH-20 column. The flow rate was 4 mL/min

Solvents	Solvent composition Acetonitrile:H_2O:TFA	Fractions collected	Volume eluted mL
**So1**	10:90:0.5	1-2	280
**So2**	20:80:0.5	3-8	1015
**So3**	30:80:0.5	9-20	2047
**So4**	40:60:0.5	21-26	1043
Solvents	Solvent composition MeOH:H_2O:TFA		Vol. eluted mL
**So5**	70:30:0.5	27-34	1137
**So6**	80:20:0.5	35-39	904
**So7**	90:10:0.5	40-50	2304

Table S5: Composition and elution volumes used for separation of XAD-7 purified extract of flowers of *Nepeta curviflora* using a 100×5 cm Sephadex LH-20 column. The flow rate was 4 mL/min

Solvents	Solvent composition<br>MeOH:H_2O:TFA	Vol. eluted<br>mL
**So1**	20:80:0.5	570
**So2**	30:70:0.5	1079
**So3**	35:65:0.5	559
**So4**	40:60:0.5	485
**So5**	45:55:0.5	348
**So6**	50:50:0.5	333
**So7**	60:80:0.5	317
**So8**	70:30:0.5	1344
**So9**	80:20:0.5	1915

Table S6: *In-vitro* anti-bacterial activity data for compound 1 and 2 and for the XAD purified materials against gram-negative bacteria. Inhibition zone diameter is in millimetre.

Compounds	Conc. mg/mL	*E. coli*	*K. pneumoniae*	*p. mirabilis*	*p. aeruginosa*
Gentamycin	6	33.0	35.0	32.3	30.7
Erythromycin	6	24.7	24.0	19.0	-
DMSO	-	-	-	-	-
XAD purified extract	6	-	-	-	-
cpd 1	6	-	-	-	-
cpd 2	6	-	-	-	-

Table S7: *In-vitro* anti-bacterial activity data for compounds 1 and 2 and for the XAD purified materials against gram positive bacteria. Inhibition zone diameter is in millimetre

Compounds	Conc. mg/mL	*S. aureus*	*M. Luteus*	*B. subtilis*	*E. faecalis*	*S. epidemidis*
Gentamycin	6	30.3	37.7	39.3	-	39.7
Erythromycin	6	44.0	40.3	-	-	32.3
DMSO	-	-	-	-	-	-
XAD purified extract	6	-	6.7	-	-	-
cpd 1	6	-	-	-	-	-
cpd 2	6	-	-	-	-	-
